# Plasmid profile analysis of *Escherichia coli* and *Salmonella enterica* isolated from pigs, pork and humans

**DOI:** 10.1017/S0950268822000814

**Published:** 2022-05-10

**Authors:** Jiratchaya Puangseree, Rangsiya Prathan, Songsak Srisanga, Sunpetch Angkittitrakul, Rungtip Chuanchuen

**Affiliations:** 1 Research Unit for Microbial Food Safety and Antimicrobial Resistance, Department of Veterinary Public Health, Faculty of Veterinary Science, Chulalongkorn University, Bangkok 10330, Thailand; 2 Center for Antimicrobial Resistance Monitoring in Food-borne Pathogens, Faculty of Veterinary Science, Chulalongkorn University, Bangkok 10330, Thailand; 3 Faculty of Veterinary Medicine, Khon Kaen University, Khon Kaen 40002, Thailand

**Keywords:** *Escherichia coli*, incompatibility group, plasmid, *Salmonella*, Thailand

## Abstract

This study aimed to determine the epidemiology and association of antimicrobial resistance (AMR) among *Escherichia coli* and *Salmonella* in Thailand. The *E. coli* (*n* = 1047) and *Salmonella* (*n* = 816) isolates from pigs, pork and humans were screened for 18 replicons including HI1, HI2, I1-*γ*, X, L/M, N, FIA, FIB, W, Y, P, FIC, A/C, T, FIIAs, F, K and B/O using polymerase chain reaction-based replicon typing. The *E. coli* (*n* = 26) and *Salmonella* (*n* = 3) isolates carrying IncF family replicons, ESBL and/or *mcr* genes were determined for FAB formula. IncF represented the major type of plasmids. Sixteen and eleven Inc groups were identified in *E. coli* (85.3%) and *Salmonella* (25.7%), respectively. The predominant replicon patterns between *E. coli* and *Salmonella* were IncK-F (23.7%) and IncF (46.2%). Significant correlations (*P* < 0.05) were observed between plasmid-replicon type and resistance phenotype. Plasmid replicon types were significantly different among sources of isolates and sampling periods. The most common FAB types between *E. coli* and *Salmonella* were F2:A-:B- (30.8%) and S1:A-:B- (66.7%), respectively. In conclusion, various plasmids present in *E. coli* and *Salmonella*. Responsible and prudent use of antimicrobials is suggested to reduce the selective pressures that favour the spread of AMR determinants. Further studies to understand the evolution of R plasmids and their contribution to the dissemination of AMR genes are warranted.

## Introduction

Antimicrobial resistance (AMR) constitutes a complex and multifaceted public health challenge that requires a board-integrated one health approach to deal with. AMR monitoring and surveillance has been established across human, animal and environmental sectors to understand the burden and ecology of the problem. As for AMR monitoring and surveillance in food-animal origin, target bacteria included commensal *Escherichia coli* and *Salmonella* [1]. Commensal *E. coli* normally live in the large intestines of humans and animals, serving as reservoirs of AMR determinants that could spread to bacterial pathogens. *Salmonella* is a food-borne zoonotic bacterial pathogen prevalent in food animals and meat; it is also frequently resistant to multiple antibiotics. Both bacteria possess a vast array of R plasmids, conjugative plasmids conferring on bacteria resistance to one or more antibiotics, that are critical positions for the spread of AMR determinants [2].

Mobile genetic element acquisition, especially plasmid, via horizontal transmission is a major route for the emergence and dissemination of AMR [3]. Transmissible R plasmids usually carry multiple genes encoding resistance to clinically relevant antibiotics and play an important role in AMR evolution and spread. Certain species-specific association plasmids exist e.g. IncX plasmids in *Salmonella* and *E. coli* [4] and IncF plasmids in Enterobacteriaceae [5]. Previous studies investigated the dynamics and diversity of AMR among humans, livestock and food of animal origin [6–8]. A variety of AMR determinants have been found to be associated with conjugative plasmids. The same genetic elements were detected in different bacterial species from different sources and locations. For example, class 1 integrons with *dfrA12-aadA2* cassette were isolated from *Salmonella* in pigs [6, 7], poultry [7, 9] and humans [6, 9]; *E. coli* in pigs [8, 10], poultry [8]; *Aeromonas hydrophila* in Nile Tilapia [11] and *Pseudomonas aeruginosa* and *Acinetobacter baumannii* in patients [12]. These findings underscore the horizontal transfer of plasmids as a major driver for AMR dissemination in Thailand and neighbouring countries.

A classical method for plasmid identification and classification is incompatibility (Inc) group testing [4]. To date, at least 27 different Inc groups of plasmids have been identified among Enterobacteriaceae [13]. Plasmids in the same Inc group share the same replication control or partitioning mechanisms and can neither coexist in the same bacterial cells nor be co-transferred [14]. The presence of bacterial strains originated from different sources but carrying plasmids of the same Inc group indicate the horizontal widespread of the plasmids with close-phylogenetic relationship. Accordingly, molecular epidemiological investigation of plasmids has been used to trace the source and potential risk of AMR spread via plasmids.

Data from molecular epidemiological analysis of plasmids will increase knowledge and understanding of plasmid diversity and transmission and benefit the development of strategic action plan to contain AMR. This study aimed to characterise the plasmid profiles in *E. coli* and *Salmonella* from pigs, pork and humans in Thailand.

## Materials and methods

### Bacterial isolates and their AMR phenotype and genotype

*E. coli* (*n* = 1047) and *Salmonella* (*n* = 816) isolates were included in this study. They originated from our previous epidemiological studies investigating AMR in healthy food animals, meat and humans during 2005–2019 [6, 9, 10, 15–18] (Table 1). The research protocols involving human subjects in these previous studies were approved by Ethics Committee of the Faculty of Medicine of Khon Kaen University (the authorisation ID, HE572136). There was no involving of the human sampling in this study, thus the ethical approval was not issued.

**Table 1.** Sources and number of *E. coli* (*n* = 1047) and *Salmonella* (*n* = 816) used in this study

Year	No. of *E. coli* isolates			Total	No. of *Salmonella* isolates			Total
	Pig	Pork	Human		Pig	Pork	Human	
2005–2010	309	–	–	309	8	104	52	164
2010–1014	123	223	103	449	67	263	85	415
2015–2019	265	24	–	289	94	143	–	237
Total	697	247	103	1047	169	510	137	816
Grand total	1047				816			

All the *E. coli* strains were isolated from rectal swabs of clinically healthy pigs (*n* = 697), pork (*n* = 247) and humans (*n* = 103) from Northern, Northeastern, Central and Western Thailand. A single colony of *E. coli* was collected from each positive sample.

The *Salmonella* isolates originated from pigs (*n* = 169), pork (*n* = 510) and humans (*n* = 137) in Northern, Northeastern and Central Thailand (Table 1). *Salmonella* was isolated as described in ISO6579:2017 [19] and serotyped using slide agglutination. A single colony of each serovar was collected from each positive sample. Rissen was the most common serovar among the *Salmonella* isolated from pigs (30.8%, 52/169) and pork (29.2%, 149/510), while *Salmonella* Stanley was the most predominant among the isolates from humans (26%, 19/137) (Table S1 in Supplementary material).

All *E. coli* and *Salmonella* isolates were previously tested for susceptibilities to nine antimicrobial agents including ampicillin (AMP), chloramphenicol (CHP), ciprofloxacin (CIP), gentamycin (GEN), streptomycin (STR), sulphamethoxazole (SMZ), tetracycline (TET), trimethoprim (TMP), colistin (COL) and phenotypically detected for extended-spectrum-betalactamese (ESBL) production [20] (Table 2). All the isolates were also screened for *mcr-1*, *mcr-2* and *mcr-3*. Ten per cent of *E. coli* and 1.5% *Salmonella* carried at least one *mcr*. The ESBL-producing *E. coli* (*n* = 155) were tested for ESBL genes and found to harbour *bla*_CTX-M_ (95.5%), *bla*_TEM_ (80.6%) and *bla*_CMY-2_ (1.3%). The *bla*_CTX-M_ group (95.2%) and *bla*_TEM_ (33.3%) were found in ESBL-producing *Salmonella* (*n* = 21) (Table 2). The relevant resistance phenotypes are indicated in the text when appropriate.

**Table 2.** AMR and ESBL production in *E. coli* (*n* = 1047) and *Salmonella* (*n* = 816) isolates that included in this study

Antimicrobial drugs/enzymes	No. of *E. coli* (%)				No. of *Salmonella* (%)			
	Pig (*n* = 697)	Pork (*n* = 247)	Human (*n* = 103)	Total (*n* = 1047)	Pig (*n* = 169)	Pork (*n* = 510)	Human (*n* = 137)	Total (*n* = 816)
AMP	623 (89.4)	200 (81.0)	62 (60.2)	885 (84.5)	148 (87.6)	395 (77.5)	89 (65.0)	632 (77.5)
CHP	423 (60.7)	83 (33.6)	17 (16.5)	523 (50.0)	25 (14.8)	147 (28.8)	83 (60.6)	255 (31.3)
CIP	220 (31.6)	8 (3.2)	7 (6.8)	235 (22.4)	0 (0)	2 (0.4)	21 (15.3)	23 (2.8)
GEN	279 (40.0)	29 (11.7)	14 (13.6)	322 (30.8)	30 (17.8)	69 (13.5)	79 (57.7)	178 (21.8)
STR	453 (65.0)	114 (46.2)	11 (10.7)	578 (55.2)	109 (64.5)	323 (63.3)	123 (89.8)	555 (68.0)
SMZ	521 (74.7)	121 (49.0)	38 (36.9)	680 (64.9)	130 (76.9)	408 (80)	103 (75.2)	641 (78.6)
TET	617 (88.5)	169 (68.4)	51 (49.5)	837 (79.9)	140 (82.8)	426 (83.5)	106 (77.4)	672 (82.4)
TMP	475 (68.1)	127 (51.4)	34 (33.0)	636 (60.7)	95 (56.2)	241 (47.3)	64 (46.7)	400 (49.0)
COL	160 (23.0)	15 (6.1)	0 (0)	175 (16.7)	2 (1.2)	7 (1.4)	0 (0)	9 (1.1)
ESBLs	140 (20.1)	7 (2.8)	8 (7.8)	155 (14.8)	2 (1.2)	19 (3.7)	0 (0)	21 (2.6)

AMP, ampicillin; CHP, chloramphenicol; CIP, ciprofloxacin; GEN, gentamycin; STR, streptomycin; SMZ, sulphamethoxazole; TET, tetracycline; TMP, trimethoprim; COL, colistin.

### Plasmid incompatibility grouping by PBRT

Plasmid incompatibility groups were identified by polymerase chain reaction (PCR)-based replicon-typing (PBRT) in all *E. coli* and *Salmonella* isolates using 18 targeting replicons using specific primers [21] (Table S2 in Supplementary material). PCR-DNA templates were prepared by the whole-cell boiling method [22]. PCRs were prepared using the Toptaq Master Mix kit (QIAGEN, Hilden, Germany) according to the manufacturer's instructions.

### Replicon sequence typing (RST)

Since IncF was the most common plasmid, the *E. coli* (*n* = 26) and *Salmonella* (*n* = 3) isolates that carried ESBL and/or *mcr* genes and IncF plasmid were tested using the RST scheme [23] (Table S2 in Supplementary material). The RST scheme included the PCR amplification of FIA, using the same primers FIA FW/FIA RV that were used in the PBRT scheme; FII, using FII FW/FII RV for *E. coli* and FIIs FW/FIIs RV for *Salmonella* and FIB, using FIB FW/FIB RV for *E. coli* and FIBs FW/FIB RV for *Salmonella*, respectively. PCR products were purified using Nucleospin gel and PCR clean up (McCherey-Nagel, Düren, Germany) and submitted to First Base Laboratories (Selangor Darul Ehsan, Malaysia) for nucleotide sequencing. The obtained sequences were analysed using the DNA-star program (DNAstar, Madison, WI) and Blast search program (https://blast.ncbi.nlm.nih.gov/Blast.cgi) and then, compared to alleles available at https://pubmlst.org/plasmid/.

### Statistical analysis

The prevalence of plasmid replicon types was analysed using Microsoft Excel. Comparisons of the associations between plasmid replicon types and AMR phenotypes were performed separately using odds ratios (OR) by SPSS version 22.0. Comparisons of the replicon type prevalence of *E. coli* and *Salmonella* from different sources and years were conducted using Fisher's exact test. A *P*-value of <0.05 was considered statistically significant. ORs and 95% confidence intervals (CIs) were calculated.

## Results

### Plasmid replicon types of *E. coli*

Sixteen replicon types (except for IncL/M and T) were identified in the *E. coli* isolates (Table 3), of which IncK replicon (60.6%, 634/1047) and IncF (48.9%, 512/1047) were most common. The HI2 (2.7%, 19/697), W (0.1%, 1/697) and X (0.1%, 1/697) replicons were limited to the pig isolates.

**Table 3.** Percentage of Inc group of plasmids of *E. coli* (*n* = 1047) and *Salmonella* (*n* = 816) isolated from pig, pork and human

Target bacteria	Category	Sub-category	No. of isolates for each replicon (%)															
			HI1	HI2	I1-*γ*	X	N	FIA	FIB	W	Y	P	FIC	A/C	FIIAs	K	B/O	F
*E. coli* (*n* = 1047)	Overall (*n* = 1047)		137 (13.1)	19 (1.8)	129 (12.3)	1 (0.1)	112 (10.7)	59 (5.6)	290 (27.7)	1 (0.1)	135 (12.9)	23 (2.2)	39 (3.7)	12 (1.1)	146 (13.9)	634 (60.6)	16 (1.5)	512 (48.9)
	By source (*n* = 1047)	Pig (*n* = 697)	120 (17.2)^a^	19 (2.7)^a^	108 (15.5)^a^	1 (0.1)^a^	93 (13.3)^a^	49 (7)^a^	240 (34.4)^a^	1 (0.1)^a^	105 (15.1)^a^	9 (1.3)^a^	19 (2.7)^a^	5 (0.7)^a^	127 (18.2)^a^	509 (73)^a^	15 (2.2)^a^	405 (58.1)^a^
		Pork (*n* = 247)	11 (4.5)^b^	0^b^	14 (5.7)^b^	0^a^	16 (6.5)^b^	5 (2)^b^	32 (13)^b^	0^a^	24 (9.7)^b^	7 (2.8)^a,b^	18 (7.3)^b^	6 (2.4)^b^	18 (7.3)^b^	106 (42.9)^b^	0^b^	73 (29.6)^b^
		Human (*n* = 103)	6 (5.8)^b^	0^a,b^	7 (6.8)^b^	0^a^	3 (2.9)^b^	5 (4.9)^a,b^	18 (17.5)^b^	0^a^	6 (5.8)^b^	7 (6.8)^b^	2 (1.9)^a,b^	1 (1)^a,b^	1 (1)^c^	19 (18.4)^c^	1 (1)^a,b^	34 (33)^b^
	By year	2007–2010 (*n* = 309)	63 (20.4)^a^	19 (6.1)^a^	86 (27.8)^a^	0^a^	46 (14.9)^a^	28 (9.1)^a^	111 (35.9)^a^	0^a^	31 (10.0)^a^	0^a^	5 (1.6)^a^	0^a^	33 (10.7)^a^	246 (79.6)^a^	11 (3.6)^a^	202 (65.4)^a^
		2011–2014 (*n* = 449)	33 (7.3)^b^	0^b^	29 (6.5)^b^	0^a^	32 (7.1)^b^	16 (3.6)^b^	70 (15.6)^b^	0^a^	46 (10.2)^a^	18 (4.0)^b^	27 (6.0)^b^	10 (2.2)^b^	24 (5.3)^b^	163 (36.3)^b^	1 (0.2)^b^	140 (31.2)^b^
		2015–2019 (*n* = 289)	41 (14.2)^a^	0^b^	14 (4.8)^b^	1 (0.3)^a^	34 (11.8)^a^	15 (5.2)^ab^	109 (37.7)^a^	1 (0.3)^a^	58 (20.1)^b^	5 (1.7)^b^	7 (2.4)^a^	2 (0.7)^ab^	89 (30.8)^c^	225 (77.9)^a^	4 (1.4)^ab^	170 (58.8)^a^
*Salmonella* (*n* = 816)	Overall (*n* = 816)		21 (2.6)	2 (0.2)	35 (4.3)	na	20 (2.5)	na	19 (2.3)	na	40 (4.9)	na	6 (0.7)	15 (1.8)	81 (9.9)	na	1 (0.1)	22 (2.7)
	By source (*n* = 816)	Pig (*n* = 169)	3 (1.8)^a,b^	0^a^	18 (10.7)^a^	na	1 (0.6)^a^	na	6 (3.6)^a^	na	34 (20.1)^a^	na	0^a^	0^a^	12 (7.1)^a^	na	0^a^	8 (4.7)^a^
		Pork (*n* = 510)	18 (3.5)^a^	2 (0.4)^a^	14 (2.7)^b^	na	6 (1.2)^a^	na	13 (2.5)^a,b^	na	4 (0.8)^b^	na	6 (1.2)^a^	9 (1.8)^a,b^	36 (7.1)^a^	na	0^a^	14 (2.7)^a^
		Human (*n* = 137)	0^b^	0^a^	3 (2.2)^b^	na	13 (9.5)^b^	na	0^b^	na	2 (1.5)^b^	na	0^a^	6 (4.4)^b^	33 (24.1)^b^	na	1 (0.7)^a^	0^b^
	By year	2005–2010 (*n* = 164)	0^a^	2 (1.2)^a^	7 (4.3)^ab^	na	15 (9.1)^a^	na	8 (4.9)^a^	na	1 (0.6)^a^	na	5 (3.0)^a^	3 (1.8)^a^	10 (6.1)^a^	na	0^a^	9 (5.5)^a^
		2011–2014 (*n* = 415)	15 (3.6)^b^	0^a^	11 (2.7)^a^	na	5 (1.2)^b^	na	10 (2.4)^ab^	na	6 (1.4)^a^	na	1 (0.2)^b^	7 (1.7)^a^	50 (12.0)^b^	na	1 (0.2)^a^	11 (2.7)^ab^
		2015–2019 (*n* = 237)	6 (2.5)^ab^	0^a^	17 (7.2)^b^	na	0^b^	na	1 (0.4)^b^	na	33 (13.9)^b^	na	0^b^	5 (2.1)^a^	21 (8.9)^ab^	na	0^a^	2 (0.8)^b^

^a,b,c^Values with different superscripts in the same column and category indicated statistical difference (*P* < 0.05) among *E. coli* or *Salmonella* from different sources or years.

na, no associations due to the lack of the corresponding replicon types.

The predominant replicon type in the human isolates was IncF (33%, 34/103), while IncK plasmids were predominant in the pigs (73%, 509/697) and pork (42.9%, 106/247) isolates. IncFIIAs (18.2%, 127/697) and K (73%, 509/697) plasmids were significantly higher (*P* < 0.05) in the pig isolates than those from other sources. The prevalence of IncHI1, I1-*γ*, N, FIB, Y, FIIAs, K and F among *E. coli* from pigs (17.2% (120/697), 15.5% (108/697), 13.3% (93/697), 34.4% (240/697), 15.1% (105/697), 18.2% (127/697), 73% (509/697) and 58.1% (405/697), respectively) were significantly higher (*P* < 0.05) than those from other sources.

**Fig. 1.**
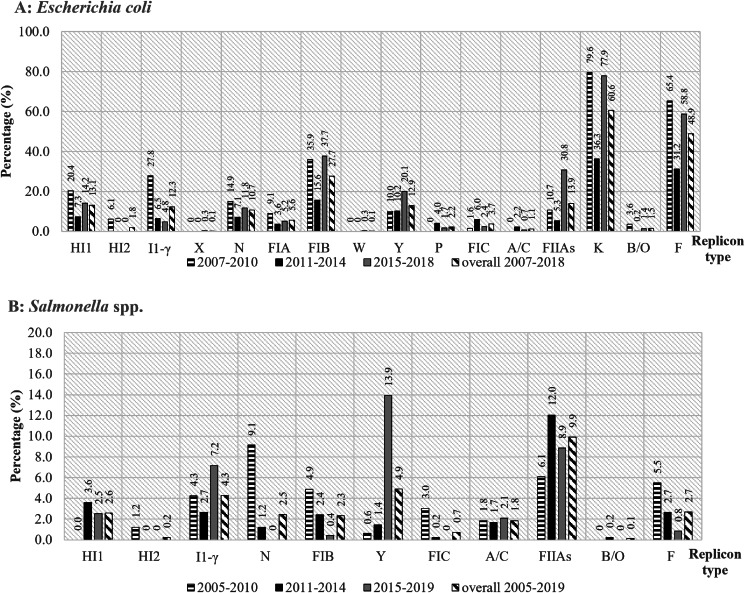
Prevalence of replicon types of (A) *E. coli* and (B) *Salmonella* sorted by year, 2007–2010 (*n* = 309, 164), 2011–2014 (*n* = 449, 415) and 2015–2019 (*n* = 289, 237), respectively.

When considering years of isolates, IncK and IncF were the most predominant replicons in all periods, 2007–2010 (79.6% (246/309) and 65.4% (202/309)), 2011–2014 (36.6% (163/449) and 31.2% (140/449)) and 2015–2019 (77.9% (225/289) and 58.8% (225/289)), respectively (Fig. 1). The IncX (0.3%, (1/289)) and W (0.3% (1/289)) plasmids were identified at a very limited rate and only in 2015–2019. The percentage of IncHI1 (20.4% (63/309), 14.2% (41/289)), N (14.9% (46/309), 11.8% (34/289)), FIB (35.9% (111/309), 37.7% (109/289)), FIIAs (10.7% (33/309), 30.8% (89/289)), K (79.6% (246/309), 77.9% (225/289)) and F (65.4% (202/309), 58.8% (170/289)) plasmids among the *E. coli* isolates during 2007–2010 and 2015–2019, respectively, were significantly higher (*P* < 0.05) than those during 2011–2014. In contrast, the presence of IncP (4.0%, 18/449) and FIC (6.0%, 27/449) plasmids from 2011 to 2014 were significantly higher than those in other years (*P* < 0.05) (Table 3).

Up to 66 replicon patterns were defined (Table 4), of which the K–F replicon pattern was most common (23.7%). Thirty replicon patterns were found in ESBL-producing *E. coli* (*n* = 155), of which I1*γ*-K–F was the most frequently found (27.3%). The *mcr*-carrying *E. coli* (*n* = 109) had 27 replicon patterns, of which K–F (18.3%) was the most common.

**Table 4.** Replicon patterns among *E. coli* (*n* = 1047) and *Salmonella* (*n* = 816)

Replicon pattern^a^	*E. coli*			*Salmonella* spp.		
	No. of isolates (%)	No. of ESBL-producing isolates (%)	No. of *mcr*-carrying isolates (%)	No. of isolates (%)	No. of ESBL-producing isolates (%)	No. of *mcr*-carrying isolates (%)
A/C	1 (0.1)	–	–	10 (4.8)	5 (23.8)	–
A/C-F	3 (0.3)	1 (0.6)	–	1 (0.5)	–	–
A/C-K	1 (0.1)	–	–	–	–	–
A/C-K-F	2 (0.2)	–	–	–	–	–
B/O	1 (0.1)	–	–	1 (0.5)	–	–
B/O-K-F	10 (1.1)	–	1 (0.9)	–	–	–
F	129 (14.4)	13 (8.4)	8 (7.3)	97 (46.2)	1 (5.3)	3 (60.0)
F-Y-K	36 (4.0)	11 (7.1)	4 (3.7)	–	–	–
HI1	8 (0.9)	2 (1.3)	2 (1.8)	10 (4.8)	8 (42.1)	1 (20.0)
HI1-F	10 (1.1)	1 (0.6)	5 (4.6)	7 (3.0)	1 (5.3)	1 (20.0)
HI1-HI2-F	4 (0.4)	–	4 (3.7)	–	–	–
HI1-HI2-K-F	15 (1.7)	7 (4.5)	11 (10.1)	–	–	–
HI1-I1-*γ*	–	–	–	4 (1.9)	4 (21.1)	–
HI1-I1-*γ*-F	2 (0.2)	–	–	–	–	–
HI1-I1-*γ*-K-B/O-F	5 (0.6)	–	5 (4.6)	–	–	–
HI1-I1-*γ*-K-F	3 (0.3)	1 (0.6)	1 (0.9)	–	–	–
HI1-K	11 (1.2)	1 (0.6)	7 (6.4)	–	–	–
HI1-K-F	24 (2.7)	2 (1.3)	6 (5.5)	–	–	–
HI1-N	5 (0.6)	–	–	–	–	–
HI1-N-A/C-K	1 (0.1)	–	–	–	–	–
HI1-N-F	8 (0.9)	3 (1.9)	3 (2.8)	–	–	–
HI1-N-K	13 (1.5)	8 (5.2)	–	–	–	–
HI1-N-K-F	11 (1.2)	2 (1.3)	–	–	–	–
HI1-N-Y	1 (0.1)	–	–	–	–	–
HI1-N-Y-F	1 (0.1)	–	–	–	–	–
HI1–N-Y-K	1 (0.1)	–	–	–	–	–
HI1-N-Y-K-F	2 (0.2)	–	–	–	–	–
HI1-Y	1 (0.1)	1 (0.6)	–	–	–	–
HI1-Y-A/C-K	1 (0.1)	1 (0.6)	–	–	–	–
HI1-Y-F	1 (0.1)	1 (0.6)	–	–	–	–
HI1-Y-K	8 (0.9)	8 (5.2)	1 (0.9)	–	–	–
HI1-Y-K-F	1 (0.1)	–	–	–	–	–
HI2	–	–	–	1 (0.5)	–	–
HI2-N	–	–	–	1 (0.5)	–	–
I1-*γ*	8 (0.9)	1 (0.6)	–	16 (7.6)	–	–
I1-A/C-F	1 (0.1)	–	1 (0.9)	–	–	–
I1-F	16 (1.8)	4 (2.6)	1 (0.9)	3 (1.4)	–	–
I1-*γ*-F-Y-P	1 (0.1)	–	–	–	–	–
I1-*γ*-K	9 (1.0)	–	–	–	–	–
I1-*γ*-K-F	61 (6.8)	42 (27.3)	4 (3.7)	–	–	–
I1-*γ*-N-K-F	1 (0.1)	–	–	–	–	–
I1-*γ*-P-F	1 (0.1)	–	–	–	–	–
I1-*γ*-Y	6 (0.7)	3 (1.9)	–	12 (5.7)	–	–
I1-*γ*-Y-A/C-K-F	1 (0.1)	–	–	–	–	–
I1-*γ*-Y-F	3 (0.3)	–	–	–	–	–
I1-*γ*-Y-K-F	3 (0.3)	2 (1.3)	1 (0.9)	–	–	–
I1-*γ*-Y-K	8 (0.9)	4 (2.6)	2 (1.8)	–	–	–
K	106 (11.9)	1 (0.6)	3 (2.8)	–	–	–
K-F	212 (23.7)	17 (11.0)	20 (18.3)	–	–	–
N	4 (0.4)	–	1 (0.9)	15 (7.1)	–	–
N-A/C	–	–	–	4 (1.9)	–	–
N-F	9 (1.0)	5 (3.2)	2 (1.8)	–	–	–
N-K	25 (2.8)	1 (0.6)	3 (2.8)	–	–	–
N-K-F	22 (2.5)	4 (2.6)	5 (4.6)	–	–	–
N-Y	1 (0.1)	–	–	–	–	–
N-Y-A/C-K	1 (0.1)	–	–	–	–	–
N-Y-K	1 (0.1)	–	–	–	–	–
N-Y-K-F	5 (0.6)	3 (1.9)	–	–	–	–
P	5 (0.6)	–	–	–	–	–
P-F	3 (0.3)	–	–	–	–	–
P-K	3 (0.3)	–	–	–	–	–
P-K-F	3 (0.3)	–	–	–	–	–
W-K-F	1 (0.1)	–	–	–	–	–
X-F	1 (0.1)	–	–	–	–	–
Y	12 (1.3)	–	3 (2.8)	28 (13.3)	–	–
Y-F	12 (1.3)	2 (1.3)	5 (4.6)	–	–	–
Y-K	21 (2.4)	2 (1.3)	–	–	–	–
Y-P-F	1 (0.1)	–	–	–	–	–
Y-P-K	2 (0.2)	–	–	–	–	–
Y-P-K-F	4 (0.4)	–	–	–	–	–
Positive at least one replicon type	893 (85.3)	154 (99.4)	109 (100)	210 (25.7)	19 (90.5)	5 (41.7)
No replicon pattern	154 (14.7)	1 (0.6)	–	606 (74.3)	2 (9.5)	7 (58.3)
Total	1047	155	109	816	21	12

a F, at least one replicon type of IncF family replicon (i.e. FIA, FIB, FIC, FIIAs and F) was found.

### Plasmid replicon types of *Salmonella*

Eleven plasmid replicon types, except for IncL/M, X, T, FIA, W, P and K were found among the *Salmonella* isolates (Table 3). Overall, IncFIIAs was the most common replicon type (9.9%, 81/816), followed by IncY (4.9%, 40/816) and IncI1-*γ* (4.3%, 35/816). The predominant replicon of *Salmonella* isolated from pigs was IncY (20.1%, 34/169), while that among the pork and human isolates were IncFIIAs (7.1% (36/510) and 24.1% (33/137), respectively). The percentage of IncHI1 in the pork isolates (3.5% (18/510)) and IncI1-*γ*, FIB, Y and F (10.7% (18/169), 3.6% (6/169), 20.1% (34/169) and 4.7% (8/169), respectively) among the pig isolates were significantly higher than those from humans (*P* < 0.05). In contrast, the prevalence of IncN, A/C and FIIAs (9.5% (13/137), 4.4% (6/137) and 24.1% (33/137), respectively) among human isolates were significantly higher than those among the pig and pork isolates (*P* < 0.05).

The predominant replicon types in each period varied. IncN (9.1%, 15/164) were the most common plasmids in 2005–2010, while that in 2011–2014 and 2015–2019 were IncFIIAs (12.0%, 50/415) and IncY (13.9%, 33/237), respectively. IncY plasmids in 2015–2019 (13.9%, 33/237) were significantly higher than that in the other periods (*P* < 0.05). The prevalence of IncN and FIC plasmids was the highest during 2005–2010 (9.1% (15/164) and 3.0% (5/164), respectively) (*P* < 0.05).

Fifteen-replicon patterns were found in *Salmonella* (Table 4). The most common replicon pattern was F (46.2%). The ESBL-producing *Salmonella* (*n* = 21) had five replicon patterns, of which HI1 (42.1%) was the most common.

### Association between replicon type and AMR phenotype in *E. coli* and *Salmonella*

Overall, the significant positive associations were more frequently observed than the negative association in both *E. coli* and *Salmonella* (Table 5).

**Table 5.** OR between the presence of replicon types and AMR or ESBL-producing *E. coli* (*n* = 1047) and *Salmonella* (*n* = 816)

Bacterial strain	Type of replicons	OR of AMR phenotype (95% CI)									
		AMP	CHP	CIP	GEN	STR	SMZ	TET	TMP	COL	ESBL producer
*E. coli*	HI1	4.96 (2.0–12.4)^a^	2.62 (1.8–3.9)^a^	5.46 (3.7–8.0)^a^	5.0 (3.4–7.3)^a^	3.7 (2.4–5.7)^a^	4.2 (2.4–7.4)^a^	12.1 (3.8–38.4)^a^	2.56 (1.7–3.9)^a^	6.83 (4.6–10.1)^a^	2.5 (1.6–3.8)^a^
	HI2	na	na	na	–	na	na	na	–	20.34 (6.7–62.1)^a^	3.36 (1.3–8.7)^a^
	I1-*γ*	–	–	0.52 (0.3–0.9)^b^	4.77 (3.2–7.0)^a^	2.5 (1.7–3.9)^a^	–	–	0.55 (0.4–0.8)^b^	–	6.33 (4.2–9.5)^a^
	N	4.95 (1.8–13.7)^a^	2.78 (1.8–4.3)^a^	3.41 (2.3–5.1)^a^	2.34 (1.6–3.5)^a^	1.83 (1.2–2.8)^a^	2.8 (1.6–4.7)^a^	3.3 (1.6–6.9)^a^	2.95 (1.8–4.8)^a^	1.79 (1.1–2.8)^a^	1.82 (1.1–2.9)^a^
	FIA	–	–	3.67 (2.2–6.3)^a^	–	2.0 (1.1–3.6)^a^	5.22 (2.1–13.2)^a^	2.61 (1.02–6.6)^a^	2.53 (1.3–4.8)^a^	–	0.19 (0.04–0.8)^b^
	FIB	3.16 (1.9–5.3)^a^	1.62 (1.2–2.1)^a^	2.0 (1.5–2.7)^a^	–	1.56 (1.2–2.1)^a^	2.15 (1.6–3.0)^a^	2.82 (1.8–4.3)^a^	1.58 (1.2–2.1)^a^	2.36 (1.7–3.3)^a^	–
	Y	3.37 (1.5–7.4)^a^	–	1.61 (1.1–2.4)^a^	1.92 (1.3–2.8)^a^	2.39 (1.6–3.6)^a^	–	2.17 (1.2–3.9)^a^	1.91 (1.3–2.9)^a^	1.93 (1.3–3.0)^a^	2.57 (1.7–4.0)^a^
	P	–	–	na	0.1 (0.01–0.7)^b^	–	–	–	–	na	na
	FIC	–	0.28 (0.1–0.6)^b^	–	–	–	–	–	–	–	–
	A/C	na	–	–	–	–	–	–	–	–	–
	FIIAs	–	–	–	0.65 (0.4–0.9)^b^	–	–	–	–	1.56 (1.02–2.3)^a^	0.51 (0.2–0.9)^b^
	K	2.38 (1.7–3.4)^a^	2.39 (1.9–3.1)^a^	1.77 (1.3–2.4)^a^	1.99 (1.5–2.6)^a^	2.2 (1.7–2.8)^a^	2.38 (1.8–3.1)^a^	3.3 (2.4–4.6)^a^	2.07 (1.6–2.7)^a^	1.47 (1.04–2.1)^a^	2.0 (1.4–2.9)^a^
	B/O	–	–	–	–	–	–	na	9.47 (1.3–72.0)^a^	3.06 (1.1–8.5)^a^	na
	F	2.17 (1.5–3.1)^a^	1.87 (1.5–2.4)^a^	1.4 (1.1–1.9)^a^	2.55 (1.9–3.3)^a^	1.57 (1.2–2.0)^a^	1.59 (1.2–2.1)^a^	3.0 (2.1–4.2)^a^	–	2.76 (1.9–3.9)^a^	3.23 (2.2–4.7)^a^
*Salmonella*	HI1	na	46.8 (6.2–350.8)^a^	na	4.04 (1.7–9.7)^a^	0.22 (0.1–0.6)^b^	na	–	–	11.9 (2.3–60.8)^a^	159.9 (52.0–491.3)^a^
	HI2	–	–	na	–	–	na	–	–	na	na
	I1-*γ*	–	–	na	4.5 (2.3–9.0)^a^	–	–	–	–	na	5.8 (1.8–18.3)^a^
	N	–	44.3 (5.9–332.7)^a^	14.4 (4.7–43.9)^a^	8.8 (3.3–23.2)^a^	–	–	–	–	na	na
	FIB	–	4.8 (1.8–13.0)^a^	na	–	na	–	na	–	–	na
	Y	0.17 (0.1–0.5)^b^	na	–	–	na	na	na	–	na	na
	FIC	0.1 (0.02–0.7)^b^	–	na	na	–	–	0.2 (0.04–0.98)^b^	–	20.1 (2.1–191.6)^a^	na
	A/C	na	14.8 (3.3–65.9)^a^	na	14.96 (4.2–53.7)^a^	na	na	na	–	na	24.5 (7.5–80.0)^a^
	FIIAs	0.15 (0.1–0.2)^b^	–	–	–	0.36 (0.2–0.6)^b^	–	0.2 (0.1–0.3)^b^	–	–	–
	F	–	4.85 (2.0–12.0)^a^	na	–	na	–	na	–	–	–

OR > 1, the resistance to the drug increased with the presence of corresponding replicon types.

OR < 1, the resistance to the drug decreased with the presence of corresponding replicon types.

^a,b^Statistically significant association (95% CI did not cross 1) between the presence of plasmids in particular Inc groups and resistant or ESBL-producing strains.

–, no statistically significant association (95% CI cross 1) between the presence of plasmids in particular Inc groups and resistant or ESBL-producing strains.

na, no OR due to the lack of the corresponding replicon types.

AMP, ampicillin; CHP, chloramphenicol; CIP, ciprofloxacin; GEN, gentamycin; STR, streptomycin; SMZ, sulphamethoxazole; TET, tetracycline; TMP, trimethoprim; COL, colistin.

In *E. coli*, IncHI1 exhibited the strongest positive associations (OR > 1) to AMP, CIP, GEN, STR and TET resistance. For other types of resistance phenotype/replicon associations, the strongest positive associations were between CHP/IncN (OR = 2.78), SMZ/FIA (OR = 5.22), TMP/B/O (OR = 9.47) and COL/HI2 (OR = 20.34). IncI1-*γ* plasmid showed the strongest positive association (OR = 6.33) to ESBL production.

As for *Salmonella*, IncHI1 displayed the strongest positive association (OR > 1) to CHP resistance (OR = 46.8) and ESBL production (OR = 159.9) (Table 5). Resistance to CIP, GEN and COL exhibited the highest positive association to IncN, A/C and FIC, respectively (OR > 1).

### Associations between replicon types in *E. coli* and *Salmonella*

Associations between each replicon type were diverse (Table 6). The significant positive association between IncFIB and B/O in *E. coli* was the strongest (OR = 41.24). The presence of IncFIB exhibited the strongest positive association with IncF (OR = 24.26), FIA (OR = 8.85) and FIC (OR = 2.23) replicons in *E. coli* only. The replicons with the strongest positive associations to IncHI1 (OR = 5.58), Y (OR = 3.77) and FIIAs (OR = 3.86) were IncN, P and K, respectively. The negative association between IncY and F replicons (OR = 0.66) was the strongest in *E. coli*.

**Table 6.** OR between each two replicon types presented in *E. coli* (*n* = 1047) and *Salmonella* (*n* = 816)

Bacterial strain	Replicon type	OR of replicon types (95% CI)													
		HI1	HI2	I1-*γ*	N	FIA	FIB	Y	P	FIC	A/C	FIIAs	K	B/O	F
*E. coli*	HI1	nd	na	–	5.58 (3.60–8.3)^a^	0.11 (0.02–0.79)^b^	1.55 (1.06–2.26)^a^	–	na	–	–	–	1.62 (1.10–2.39)^a^	3.10 (1.06–9.05)^a^	–
	HI2	na	nd	na	na	na	na	na	na	na	na	na	–	na	na
	I1-*γ*	–	na	nd	0.06 (0.01–0.41)^b^	–	–	–	–	–	–	0.22 (0.09–0.55)^b^	1.65 (1.11–2.47)^a^	3.31 (1.14–9.73)^a^	3.08 (2.05–4.62)^a^
	N	5.58 (3.60–8.3)^a^	na	0.06 (0.01–0.41)^b^	nd	–	0.54 (0.32–0.89)^b^	–	na	–	–	–	2.00 (1.28–3.11)^a^	na	–
	FIA	0.11 (0.02–0.79)^b^	na	–	–	nd	8.85 (4.84–16.18)^a^	–	–	–	–	–	–	–	3.96 (2.12–7.43)^a^
	FIB	1.55 (1.06–2.26)^a^	na	–	0.54 (0.32–0.89)^b^	8.85 (4.84–16.18)^a^	nd	–	–	2.32 (1.22–4.42)^a^	–	–	1.34 (1.01–1.78)^a^	41.24 (5.42–313.65)^a^	24.26 (15.43–38.14)^a^
	Y	–	na	–	–	–	–	nd	3.77 (1.57–9.06)^a^	–	–	1.67 (1.05–2.65)^a^	1.64 (1.11–2.43)^a^	na	0.66 (0.46–0.96)^b^
	P	na	na	–	na	–	–	3.77 (1.57–9.06)^a^	nd	–	na	–	–	na	–
	FIC	–	na	–	–	–	2.32 (1.22–4.42)^a^	–	–	nd	na	–	–	na	2.15 (1.09–4.23)^a^
	A/C	–	na	–	–	–	–	–	na	na	nd	na	–	na	–
	FIIAs	–	na	0.22 (0.09–0.55)^b^	–	–	–	1.67 (1.05–2.65)^a^	–	–	na	nd	3.86 (2.45–6.10)^a^	na	–
	K	1.62 (1.10–2.39)^a^	–	1.65 (1.11–2.47)^a^	2.00 (1.28–3.11)^a^	1.19 (0.69–2.06)^a^	1.34 (1.01–1.78)^a^	1.64 (1.11–2.43)^a^	–	–	–	3.86 (2.45–6.10)^a^	nd	9.98 (1.31–75.87)^a^	1.59 (1.24–2.05)^a^
	B/O	3.10 (1.06–9.05)^a^	na	3.33 (1.14–9.73)^a^	na	–	41.24 (5.42–313.65)^a^	na	na	na	na	na	9.98 (1.31–75.87)^a^	nd	16.12 (2.12–122.46)^a^
	F	–	na	3.08 (2.05–4.62)^a^	–	3.96 (2.12–7.43)^a^	24.26 (15.43–38.14)^a^	0.66 (0.46–0.96)^b^	–	2.15 (1.09–4.23)^a^	–	–	1.59 (1.24–2.05)^a^	16.12 (2.12–122.46)^a^	nd
*Salmonella*	HI1	nd	na	5.80 (1.84–18.26)^a^	na	na	na	na	na	na	na	4.87 (1.91–12.45)^a^	na	na	na
	HI2	na	nd	na	41.84 (2.52–694.23)^a^	na	na	na	na	na	na	na	na	na	na
	I1-*γ*	5.80 (1.84–18.26)^a^	na	nd	na	na	–	14.03 (6.35–31.02)^a^	na	na	na	–	na	na	–
	N	na	41.84 (2.52–694.23)^a^	na	nd	na	na	na	na	na	17.84 (5.13–62.07)^a^	na	na	na	na
	FIB	na	na	–	na	na	nd	na	na	na	–	na	na	na	na
	Y	na	na	14.03 (6.35–31.02)^a^	na	na	na	nd	na	na	na	na	na	na	na
	FIC	na	na	na	na	na	na	na	na	nd	na	–	na	na	na
	A/C	na	na	na	17.84 (5.13–62.07)^a^	na	–	na	na	na	nd	na	na	na	–
	FIIAs	4.87 (1.91–12.45)^a^	na	–	na	na	na	na	na	–	na	nd	na	na	na
	F	na	na	–	na	na	na	na	na	na	–	na	na	na	nd

OR > 1, the presence of the replicon type increased with the presence of corresponding replicon types.

OR < 1, the presence of the replicon type decreased with the presence of corresponding replicon types.

a,b Statistically significant association (95% CI did not cross 1) between the presence of plasmids in particular Inc groups and resistant or ESBL-producing strains.

–, no statistically significant association (95% CI cross 1) between the presence of plasmids in particular Inc groups and resistant or ESBL-producing strains.

na, no OR due to the lack of the corresponding replicon types.

nd, no OR because the statistics could not be determined.

In *Salmonella*, the strongest positive association was observed between IncHI2 and IncN (OR = 41.84). IncHI1 was positively associated with IncI1-*γ* (OR = 5.80) and FIIAs (OR = 4.87). The positive associations were additionally detected for IncI1-*γ*/IncY (OR = 14.03) and IncA/C/IncN (OR = 17.84).

### Replicon sequence types of *E. coli* and *Salmonella* carrying *bla* and/or *mcr*

Twenty-six ESBL-producing *E. coli* from pigs (*n* = 11), pork (*n* = 8) and humans (*n* = 7) and three *Salmonella* from a pig (*n* = 1) and pork (*n* = 2) were further subtyped using RST. Seven allele numbers of FII replicon including F-, F46, F18, F2, F29, F100 and S1 were identified. Three alleles including A-, A1,6 and A5,6 were detected in the FIA allele, while seven alleles (i.e. B-, B1, B20, B10, B40, B24 and B13) were observed in the FIB allele. The S1 allele was identified in two *Salmonella* carrying FIIs replicon. Thirteen FAB formulas were assigned (Table 7), of which the most common FAB formula between *E. coli* and *Salmonella* were F2:A-:B- (26.9%, 7/26) and S1:A-:B- (66.7%, 2/3), respectively.

**Table 7.** Replicon sequence types of Inc F of *E. coli (n* = 26) and *Salmonella* (*n* = 3)

Species	Strain name	Regions^a^	Provinces^b^	Sources	Year	Resistance genes	Allele number for replicon			FAB formula^d^
							FII, FIIs^c^	FIA	FIB	
*E. coli*	CREM 10	N	CRI	Pork	2016–2017	*bla*_CTX-M_, *bla*_TEM_	F46	–	–	F46:A-:B-
	CRES 14	N	CRI	Pig	2016–2017	*bla*_CTX-M_, *bla*_TEM_	F46	–	–	F46:A-:B-
	CRES 7	N	CRI	Pig	2016–2017	*mcr1*	F46	–	B20	F46:A-:B20
	FpCa1	W	RBR	Pig	2015	*bla*_CTX-M_, *bla*_TEM_, *mcr1*	F46	–	B20	F46:A-:B20
	FpEa24	W	RBR	Pig	2016–2017	*bla*_CTX-M_, *bla*_TEM_, *mcr1*	F46	–	B20	F46:A-:B20
	NK 253	NE	NKI	Human	2013–2014	*bla*_CTX-M_	F46	–	B20	F46:A-:B20
	CRES 20	N	CRI	Pig	2016–2017	*mcr3*	F18	–	B1	F18:A-:B1
	MH 95	NE	MDH	Pork	2013–2014	*bla*_CTX-M_, *bla*_TEM_	F18	–	B1	F18:A-:B1
	SaEM 37	E	SKW	Pork	2016–2017	*mcr1*	F18	–	B1	F18:A-:B1
	E405	NE	NMA	Pig	2007–2008	*bla*_CTX-M_, *bla*_TEM_, *mcr3*	F2	–	–	F2:A-:B-
	MH 70	NE	MDH	Human	2013–2014	*bla*_CTX-M_	F2	–	–	F2:A-:B-
	SaEM 19	E	SKW	Pork	2016–2017	*bla*_CTX-M_, *bla*_TEM_	F2	–	–	F2:A-:B-
	SaEM 29	E	SKW	Pork	2016–2017	*bla*_CTX-M_	F2	–	–	F2:A-:B-
	SaES 22	E	SKW	Pig	2016–2017	*bla*_CTX-M_, *bla*_TEM_, *mcr3*	F2	–	–	F2:A-:B-
	NK 261	NE	NKI	Human	2013–2014	*bla*_CTX-M_, *bla*_TEM_	F2	–	–	F2:A-:B-
	NK 262	NE	NKI	Human	2013–2014	*bla*_CTX-M_, *bla*_TEM_	F2	–	–	F2:A-:B-
	E431	W	RBR	Pig	2007–2008	*mcr2, mcr3*	F2	–	B40	F2:A-:B40
	MH 227	NE	MDH	Human	2013–2014	*bla*_CTX-M_	F29	–	B10	F29:A-:B10
	NK 276	NE	NKI	Human	2013–2014	*bla*_CTX-M_	F46	–	B24	F46:A-:B24
	PLCa 7	NE	NMA	Pig	2015	*bla*_CTX-M_, *bla*_TEM_, *mcr1*	F2	–	B20	F2:A-:B20
	PLEa 14	NE	NMA	Pig	2015	*bla*_CTX-M_, *bla*_TEM_, *mcr1*	F100	–	B13	F100:A-:B13
	SaES 46	E	SKW	Pig	2016–2017	*mcr1*	F18	A5, A6^e^	B1	F18:A5,6:B1
	NK 267	NE	NKI	Human	2013–2014	*bla*_TEM_	–	A1, A6^e^	B1	F-:A1,6:B1
	CREM 48	N	CRI	Pork	2016–2017	*bla*_CTX-M_, *bla*_TEM_	–	–	B24	F-:A-:B24
	SaEM 15	E	SKW	Pork	2016–2017	*bla*_CTX-M_, *bla*_TEM_	–	–	B24	F-:A-:B24
	SaEM 57	E	SKW	Pork	2016–2017	*bla*_CTX-M_, *bla*_TEM_	–	–	B24	F-:A-:B24
*S. weltevreden*	MH 178.1	NE	MDH	Pork	2013–2014	*bla*_CTX-M14_	S1	–	–	S1:A-:B-
*S. yalding*	NSM 11.3	NE	NKI	Pork	2016–2017	*mcr1*	S1	–	–	S1:A-:B-
*S. anatum*	CRSS 28.1	N	CRI	Pig	2016–2017	*bla*_CTX-M_, *bla*_TEM_, *mcr3*	F2	–	–	F2:A-:B-

a N, Northern; NE, North-eastern; W, West; E, East.

b CRI, Chiangrai; RBR, Ratchaburi; NKI, Nongkhai; MDH, Mukdaharn; SKW, Sakaew; NMA, Nakornratchsrima.

c Both sequences of FII and FIIs were identified to be allele F.

d FAB formula was the combination of the sequence type of FII or FIIs:FIA:FIB.

e Exactly matched to more than one reference.

F46:A-:B20 was the FAB formula shared in four *E. coli* isolates (15.4%, 4/26) from pigs (*n* = 3) and one human. F18:A-:B1 was in the *E. coli* isolates (11.5%, 3/26) from pig (*n* = 1) and pork (*n* = 2). While F-:A-:B24 was found in the *E. coli* strains (11.5%, 3/26) isolated from pork (*n* = 3). Two different FAB formulas, S1:A-:B- and F2:A-:B-, were assigned for plasmid in the *Salmonella* isolates.

## Discussion

The *E. coli* and *Salmonella* isolates in this study originated from clinically healthy pigs, pork and humans previously collected across geographical regions over a long sampling period. It is expected that only healthy animals are slaughtered for human consumption, but their healthy appearance does not guarantee the absence of resistant bacteria. Antimicrobials may be administered to the animals prior to slaughtering for infection treatment, disease prevention or growth promotion and such antimicrobial use could result in AMR acquisition in commensal bacteria and pathogens. Antimicrobial susceptibilities and determinants were investigated among the isolates in this collection. However, they have not been thoroughly investigated for resistance plasmids, despite their important role in resistance traits and resistance gene dissemination.

Until now, most studies of plasmid Inc groups have been based on the resistance genes identified. Due to the lack of wide screening reports on Inc groups, a direct comparison is rather difficult. In this study, IncK was the most frequently plasmid replicon type present in *E. coli* (60.6%) from pigs, pork and humans. Currently, there are two IncK plasmid subtypes identified, including IncK1, that are commonly found in a variety of mammals, and IncK2 that were predominantly found in poultry [24]. While studies of the Inc group are widely available for the *E. coli* isolates from pigs and pork, there is still very limited research covering IncK plasmids. Most IncK studies were conducted in the isolates of humans and poultry originally from European countries [25, 26]. In addition, the absence of IncK in the *Salmonella* isolates in this study supported a previous study demonstrating that some replicon types are specific to certain bacterial hosts [27].

When considering the sampling period of *E. coli*, IncK plasmid was continuously predominant from 2007 to 2019. In contrast, the prevalence of most of the others fluctuated. For example, HI1, N, FIA, FIB, FIIAs, K, B/O and F decreased from 2011 to 2014 and increased between 2015 and 2019. The opposite trend was observed for P, FIC and A/C. Factors that affect the maintenance of some plasmids in each period remain unclear. These changes may be involved in different sampling locations and antimicrobial use. However, the phenomenon was not obvious in *Salmonella*, and this could be due to the limited replicon type observed. In addition, many plasmids of the same Inc group were found in the *E. coli* isolates from pigs, pork and humans, indicating the circulation of the plasmids in different sectors.

The PBRT primers used for the detection of IncI1 in this study cannot differentiate IncI1 and IncI-*γ* [28]. Therefore, the IncI1-*γ* type was used to describe the results obtained. In this study, the coexistence of IncI1-*γ* type and IncHI1 was observed in *Salmonella* (OR > 1), in agreement with a previous study conducted on multidrug resistance (MDR) *Salmonella* Typhi [29]. Most *Salmonella* from pigs carried IncY replicon, in line with a previous report [30]. In addition, IncT and IncW plasmids were unidentified among the isolates in this study. This agrees with the notion that IncT and IncW are rarely detected among bacteria in the Enterobacteriaceae family in recent decades [31, 32].

IncL/M, a broad host-range plasmid, was not detected in this study. The L and M plasmids were mistakenly classified together into an incompatibility group due to their high DNA homology and later, they were genetically differentiated to two different groups [33]. Therefore, the absence of IncL/M plasmid in this study may be a false-negative result due to PCR primers used [21]. Simultaneously, IncX was absent in *Salmonella*. The limited detection of IncX plasmids may be attributable to the uncovered typing scheme. The PCR primers of the PBRT scheme used in this study were specific to IncX2. However, IncX plasmids are diverse and at least nine types of IncX (i.e. X1 to X9) have been identified worldwide [34]. Therefore, the detection capacity of the IncX plasmid family should be expanded to enhance the identification and typing of novel AMR-related plasmids in Enterobacteriaceae.

It is important to observe that the same Inc plasmids are shared among the *E. coli* and *Salmonella* isolates that originated from different sources (e.g. pigs, pork and humans). Even though the direction of gene flow between different hosts was not investigated, such observations indicate the circulation of plasmids between different hosts.

Multiple plasmids of different Inc groups were found in the same bacterial host strain in this study (Table 4). Since several AMR genes are plasmid mediated and a plasmid could carry several AMR genes, the presence of multiple plasmids agreed with the MDR phenotypes observed. The association between resistance phenotypes and replicon types varied. The significant-positive associations between resistance phenotype and replicon types were commonly observed, highlighting the important role of plasmids in the dissemination of AMR genes in *E. coli* and *Salmonella* in this study. IncHI1 plasmids in *E. coli* exhibited the strongest association with increased resistance rates to AMP, GEN, STR and TET resistance (OR > 1), suggesting the existence of corresponding resistance genes on the plasmid of this replicon type. In *Salmonella*, IncHI1 plasmid was strongly associated with CHP resistance (OR = 46.8), inconsistent with a previous study where the strong positive correlation of IncHI1 plasmids to AMP, TMP, SMZ, STR and TET resistance was demonstrated in the pathogen [29]. This discrepancy may be from the effects of different antimicrobial-selective pressure in the environment of the bacterial isolates.

Persistent resistance to chloramphenicol after the ban on its use in food-producing animals has been observed in several countries [35–37]. It was linked to co-selection caused by using other antibiotics, of which their resistance genes co-localised on the same plasmid with chloramphenicol-resistance genes. In this study, the chloramphenicol resistance rate in *E. coli* was significantly correlated to IncN (OR = 2.78). This plasmid replicon type was positively associated with resistance to the commonly used antimicrobials including AMP, GEN, STR, SMZ, TET, TMP and COL. In *Salmonella*, in addition to CHP resistance, IncHI1 plasmid was strongly associated with GEN and COL resistance and ESBL production. Such positive associations indicate the possible co-localisation on the same plasmids of the resistance genes and serve as evidence that the selective pressure imposed by the use of other antimicrobials commonly used in food animals could promote the co-selection of chloramphenicol-resistant bacteria after the ban. However, further studies to analyse the plasmid context are suggested to confirm the co-localisation of AMR genes on the same plasmid.

Conversely, negative correlations were observed between some resistance genes and replicon types. For example, IncY in *Salmonella* was significantly associated with reduced frequencies of AMP resistance (OR = 0.17). Similarly, IncFIC (OR = 0.2) and FIIAs (OR = 0.2) plasmids were significantly associated with a reduced prevalence of tetracycline resistance. This indicates that these plasmids do not frequently carry resistance genes for these tested antibiotics. Besides, non-plasmid-borne mechanisms (e.g. chromosomally encoded genes, chromosomal mutations) may present and contribute to antibiotic resistance in these bacteria [38].

Strong positive associations were observed between CIP and IncHI1 plasmids in *E. coli* (OR = 5.46) and IncN plasmid in *Salmonella* (OR = 14.4). The high quinolone resistance level in bacteria is mediated by chromosomal mutations that alter drug targets and reduce the intracellular concentration of quinolones. The presence of plasmid-mediated quinolone resistance (PMQR) genes provides low-level resistance, not exceeding the clinical breakpoint for susceptibility. However, PMQR genes facilitate higher levels of quinolone resistance if a plasmid carries two or more PMQR genes [39].

In this study, colistin resistance exhibited a strong positive association with IncHI2 (OR = 20.34) and IncHI1 (OR = 6.83) in *E. coli* and IncFIC (OR = 20.1) and IncHI1 (OR = 11.9) in *Salmonella*, in agreement with a previous study [40]. Colistin-resistance encoding genes were previously found on plasmids of several replicon types including IncI2, HI1, HI2, X4, P, F and Y [41]. A previous study revealed that the IncI2 replicon was the most common plasmid carrying colistin resistance gene in *E. coli* isolated from poultry, food and humans. However, this was not the case for this study [42].

ESBL genes are usually plasmid-borne. In this study, ESBL production showed the strongest positive association with IncI1-*γ* plasmid (OR = 6.33) in *E. coli* and IncHI1 plasmid (OR = 159.9) in *Salmonella* (Table 5). This indicates the possible localisation of ESBL genes on these plasmid replicon types, in agreement with a previous study in *E. coli* [43] and *Salmonella* [29], respectively. This was supported by the observation that the *bla*_CTX-M14_-carrying *Salmonella* from pork (*n* = 4) in this study was positive for IncI1-*γ* and HI1 plasmids (Table 4). Almost all *bla*_CTX-M_-carrying IncI1-*γ*-positive isolates also contained both IncF and IncK plasmids (43/57, 75.4%). When considering ESBL genes, most *bla*_CTX-M_-carrying *E. coli* (106/155, 68.3%) were positive for IncK plasmid, in agreement with a previous study in Europe [44]. Since these isolates harboured multiple plasmids, the location of *bla*_CTX-M_ was uncertain and could be further investigated by plasmid characterisation.

The presence of genes encoding ESBLs and colistin resistance were presented in previous study that associated with IncF family plasmids in Enterobacteriaceae [45]. In this study, the IncF family replicon, including FIA, FIB, FIC, FIIAs and F was the most common in both *E. coli* and *Salmonella* strains. Of all the 13 FAB formulas obtained, the most common FAB formula of *E. coli* was F2:A-:B- as previously observed in many studies [46, 47]. F plasmid belonging to F46:A-:B20 was identified in the *E. coli* isolates from pigs and humans. This plasmid was previously reported in *Salmonella* Typhimurium from a patient in Taiwan [48]. The F18:A-:B1 plasmid was also found in *E. coli* from pigs and pork. This plasmid was previously found in *E. coli* from poultry [46]. The same FAB formula of IncF plasmid was found among the strains from different pigs, pork and humans from various locations, indicating that the particular plasmids circulate in the food chain. Further studies are suggested to investigate if the circulation was due to horizontal transfer of the plasmid or the bacterial strain dissemination.

In summary, the results revealed a variety of plasmids distributed in pigs, pork and humans in Thailand. Plasmids were strongly associated with various resistance phenotypes. Multiple plasmids were found in the same host strain, and their major role in the spread of AMR was emphasised. Plasmid analysis serves as an epidemiological marker for AMR surveillance. To the best of our knowledge, this is the first report of plasmid replicon types among *E. coli* and *Salmonella* from pigs, pork and humans in Thailand. The findings of the replicon type in this study form a basis for future studies to explore the possible methodology to counteract horizontal transfer of plasmids.

## Data Availability

The data that support the findings of this study are available from the corresponding author on reasonable request.

## Supplementary material

For supplementary material accompanying this paper visit http://doi.org/10.1017/S0950268822000814. click here to view supplementary material
