# Supplementary material for: Plasmid profile analysis of Escherichia coli and Salmonella enterica isolated from pigs, pork and humans
Source: Epidemiol Infect. 2022 May 10;150:e110. doi: 10.1017/S0950268822000814 (PMC9214845; doi:10.1017/S0950268822000814)
Supplement: Supplementary file 1 [file S0950268822000814sup001.docx]

**Supplemental**

**Table 1.** Serovars of the *Salmonella* from different sources including pigs, pork and humans that included in this study (n=816)

| *Salmonella* serovars | No. (%) | | |
| --- | --- | --- | --- |
|  | Pig (n=169) | Pork (n=510) | Human (n=137) |
| Agona |  | 1 (0.2) | - |
| Albany | 1 (0.6) | 3 (0.6) | - |
| Anatum | 11 (6.5) | 110 (21.6) | 8 (5.8) |
| Augustenborg | 1 (0.6) | 1 (0.2) | - |
| Baiboukoum | - | 1 (0.2) | - |
| Bardo | - | - | 1 (0.7) |
| Bovismorbificans | - | 4 (0.8) | 2 (1.5) |
| Bradford | - | 1 (0.2) | - |
| Braenderup | 1 (0.6) | - | - |
| Brunei | - | - | 1 (0.7) |
| Calabar | - | 1 (0.2) | - |
| Coeln | - | 1 (0.2) | - |
| Corvallis | - | 5 (1.0) | 9 (6.6) |
| Cuckmere | - | 1 (0.2) | - |
| Derby | 2 (1.2) | 4 (0.8) | 3 (2.2) |
| Eastbourne | 1 (0.6) | - | - |
| Enteritidis | - | - | 9 (6.6) |
| Fareham | 4 (2.4) | 2 (0.4) | - |
| Fulda | - | 1 (0.2) | - |
| Give | 1 (0.6) | 9 (1.8) | 2 (1.5) |
| Hayindogo | - | 3 (0.6) | - |
| Huettwillen | 1 (0.6) | - | - |
| Hvittingfoss | - | 2 (0.4) | 2 (1.5) |
| Kedougou | 13 (7.7) | 21 (4.1) | 14 (10.2) |
| Kingston | 1 (0.6) | - | - |
| Langensalza | - | 1 (0.2) | - |
| Lexington | - | - | 4 (2.9) |
| Lille | - | - | 1 (0.7) |
| Muenster | - | 1 (0.2) | - |
| Newport | - | - | 4 (2.9) |
| Norwich | - | 2 (0.4) | - |
| Orion | 1 (0.6) | - | - |
| Panama | - | 5 (1.0) | 6 (4.4) |
| Paratyphi | - | 2 (0.4) | - |
| ParatyphiB | - | 1 (0.2) | - |
| Rideau | 1 (0.6) | 4 (0.8) | - |
| Rissen | 52 (30.8) | 149 (29.2) | 4 (2.9) |
| Saintpaul | 2 (1.2) | 21 (4.1) | 1 (0.7) |
| Sanktmarx | 5 (3.0) | 10 (2.0) | - |
| Sao | 12 (7.1) | 9 (1.8) | - |
| Schwarzengrund | 2 (1.2) | - | 1 (0.7) |
| Senftenberg | - | 1 (0.2) | 1 (0.7) |
| Ser 1,4,5,12 | - | - | 2 (1.5) |
| Ser 4,12 | - | - | 1 (0.7) |
| Ser 4,12:i | - | - | 1 (0.7) |
| Ser 4,21 | - | - | 1 (0.7) |
| Ser 4,5,12:1 | - | - | 1 (0.7) |
| Ser 4,5,12:b:- | - | - | 1 (0.7) |
| Ser 9,12:1,5 | - | - | 1 (0.7) |
| Serembah | - | 1 (0.2) | - |
| Singapore | - | 2 (0.4) | - |
| Stanley | 5 (3.0) | 33 (6.5) | 26 (19.0) |
| Typhimurium | 43 (25.4) | 62 (12.2) | 8 (5.8) |
| Urbana | - | 2 (0.4) | - |
| Vejle | - | 1 (0.2) | - |
| Virchow | - | 1 (0.2) | 3 (2.2) |
| Weltevreden | 8 (4.7) | 24 (4.7) | 19 (13.9) |
| Worthington | 1 (0.6) | 3 (0.6) | - |
| Yalding | - | 4 (0.8) | - |

**Table 2.** Primers used in this study

| PCR-reaction | | Inc  group | Name | Sequence of primers | Amplicon size (bp) | Reference |
| --- | --- | --- | --- | --- | --- | --- |
| PBRT | Multiplex 1 | HI1 | HI1 FW | 5’-GGAGCGATGGATTACTTCAGTAC-3’ | 471 | [17] |
|  |  |  | HI1 RV | 5’-TGCCGTTTCACCTCGTGAGTA-3’ |  |  |
|  |  | HI2 | HI2 FW | 5’-TTTCTCCTGAGTCACCTGTTAACAC-3’ | 644 |  |
|  |  |  | HI2 RV | 5’-GGCTCACTACCGTTGTCATCCT-3’ |  |  |
|  |  | I1-γ | I1-γ FW | 5’-CGAAAGCCGGACGGCAGAA-3’ | 139 |  |
|  |  |  | I1-γ RV | 5’-TCGTCGTTCCGCCAAGTTCGT-3’ |  |  |
|  | Multiplex 2 | X | X FW | 5’-AACCTTAGAGGCTATTTAAGTTGCTGAT-3’ | 376 | [17] |
|  |  |  | X RV | 5’-TGAGAGTCAATTTTTATCTCATGTTTTAGC-3’ |  |  |
|  |  | L/M | L/M FW | 5’-GGATGAAAACTATCAGCATCTGAAG-3’ | 785 |  |
|  |  |  | L/M RV | 5’-CTGCAGGGGCGATTCTTTAGG-3’ |  |  |
|  |  | N | N FW | 5’-GTCTAACGAGCTTACCGAAG-3’ | 559 |  |
|  |  |  | N RV | 5’-GTTTCAACTCTGCCAAGTTC-3’ |  |  |
|  | Multiplex 3 | FIA | FIA FW | 5’-CCATGCTGGTTCTAGAGAAGGTG-3’ | 462 | [17] |
|  |  |  | FIA RV | 5’-GTATATCCTTACTGGCTTCCGCAG-3’ |  |  |
|  |  | FIB | FIB FW | 5’-GGAGTTCTGACACACGATTTTCTG-3’ | 702 |  |
|  |  |  | FIB RV | 5’-CTCCCGTCGCTTCAGGGCATT-3’ |  |  |
|  |  | W | W FW | 5’-CCTAAGAACAACAAAGCCCCCG-3’ | 242 |  |
|  |  |  | W RV | 5’-GGTGCGCGGCATAGAACCGT-3’ |  |  |
|  | Multiplex 4 | Y | Y FW | 5’-AATTCAAACAACACTGTGCAGCCTG-3’ | 765 | [17] |
|  |  |  | Y RV | 5’-GCGAGAATGGACGATTACAAAACTTT-3’ |  |  |
|  |  | P | P FW | 5’-CTATGGCCCTGCAAACGCGCCAGAAA-3’ | 534 |  |
|  |  |  | P RV | 5’-TCACGCGCCAGGGCGCAGCC-3’ |  |  |
|  |  | FIC | FIC FW | 5’-GTGAACTGGCAGATGAGGAAGG-3’ | 262 |  |
|  |  |  | FIC RV | 5’-TTCTCCTCGTCGCCAAACTAGAT-3’ |  |  |
|  | Multiplex 5 | A/C | A/C FW | 5’-GAGAACCAAAGACAAAGACCTGGA-3’ | 465 | [17] |
|  |  |  | A/C RV | 5’-ACGACAAACCTGAATTGCCTCCTT-3’ |  |  |
|  |  | T | T FW | 5’-TTGGCCTGTTTGTGCCTAAACCAT-3’ | 750 |  |
|  |  |  | T RV | 5’-CGTTGATTACACTTAGCTTTGGAC-3’ |  |  |
|  |  | FIIAs | FIIAs FW | 5’-CTGTCGTAAGCTGATGGC-3’ | 270 |  |
|  |  |  | FIIAs RV | 5’-CTCTGCCACAAACTTCAGC-3’ |  |  |
|  | Simplex F | F | F_repB_ FW | 5’-TGATCGTTTAAGGAATTTTG-3’ | 270 | [17] |
|  |  |  | F_repB_ RV | 5’-GAAGATCAGTCACACCATCC-3’ |  |  |
|  | Simplex K | K | K/B FW | 5’-GCGGTCCGGAAAGCCAGAAAAC-3’ | 160 | [17] |
|  |  |  | K RV | 5’-TCTTTCACGAGCCCGCCAAA-3’ |  |  |
|  | Simplex B/O | B | B/O RV | 5’-TCTGCGTTCCGCCAAGTTCGA-3’ | 159 | [17] |
| RST |  | F | FII FW | 5’-CTGATCGTTTAAGGAATTTT-3’ | 258-262 | [19] |
|  |  |  | FII RV | 5’-CACACCATCCTGCACTTA-3’ |  |  |
|  |  |  | FIB FW^a^ | 5’-TCTGTTTATTCTTTTACTGTCCAC-3’ | 683 | [19] |
|  |  |  | FIBs FW^b^ | 5’-TGCTTTTATTCTTAAACTATCCAC-3’ | 683 | [19] |
|  |  |  | FIIs FW^c^ | 5’-CTAAAGAATTTTGATGGCTGGC-3’ | 259-260 | [19] |
|  |  |  | FIIs RV^c^ | 5’-CAGTCACTTCTGCCTGCAC-3’ |  |  |

^a^ Use in a pair with FIB RV for detection of FIB replicon in *E. coli* isolates.

^b^ Use in a pair with FIB RV for detection of FIB or FII replicon in *Salmonella* isolates.

^c^ Use for detection of FII replicon in *Salmonella* isolates.
